# Birt-Hogg-Dubé syndrome detected incidentally by asymptomatic bilateral pneumothorax in health screening: a case of a young Japanese woman

**DOI:** 10.1186/s40792-015-0014-8

**Published:** 2015-02-18

**Authors:** Kentaro Miura, Ryoichi Kondo, Makoto Kurai, Keiko Ishii

**Affiliations:** Department of Thoracic Surgery, National Hospital Organization Matsumoto Medical Center, 811 Kotobukitoyooka, Matsumoto, 399-0021 Japan; Department of Division of Diagnostic Pathology, Okaya Municipal Hospital, Okaya, 394-8512 Japan

**Keywords:** Birt-Hogg-Dubé syndrome, Family history, Spontaneous pneumothorax

## Abstract

Birt-Hogg-Dubé syndrome (BHD) is an autosomal dominant disease caused by mutations of germline folliculin (FCLN) mapped in the chromosome 17p11.2 region. BHD commonly accompanies renal tumors, fibrofolliculomas, multiple pulmonary cysts, and spontaneous pneumothorax. We report a case of a young Japanese woman in whom asymptomatic bilateral pneumothorax was found incidentally in a health screening, which led to the diagnosis of BHD. She had developed neither renal tumors nor fibrofolliculomas. However, her father, uncle, and aunt also experienced pneumothorax. In Japan, BHD is not yet well known because skin-related symptoms of fibrofolliculomas are sometimes absent unlike in most cases in Europe and the United States. On the basis of this case, we propose that BHD should be considered at the time of pneumothorax examination.

## Background

Birt-Hogg-Dubé syndrome (BHD) is an autosomal dominant disease caused by mutations of germline folliculin (FCLN) mapped in the chromosome 17p11.2 region, and it commonly accompanies renal tumors; skin conditions such as fibrofolliculomas, trichodiscoma, and acrochordon; multiple pulmonary cysts; and spontaneous pneumothorax [1-3].

BHD is well known and reported most often in Europe and the United States because cases in these regions have characteristic skin symptoms. However, in East Asia, including Japan, BHD was relatively difficult to recognize until 2002, when FCLN gene detection became possible because less patients with BHD have skin-related symptoms. We report a case of a young Japanese woman in whom asymptomatic bilateral pneumothorax was found incidentally in a health screening, which led to the diagnosis of BHD, and she experienced no skin-related symptoms.

## Case presentation

**Figure 1:**
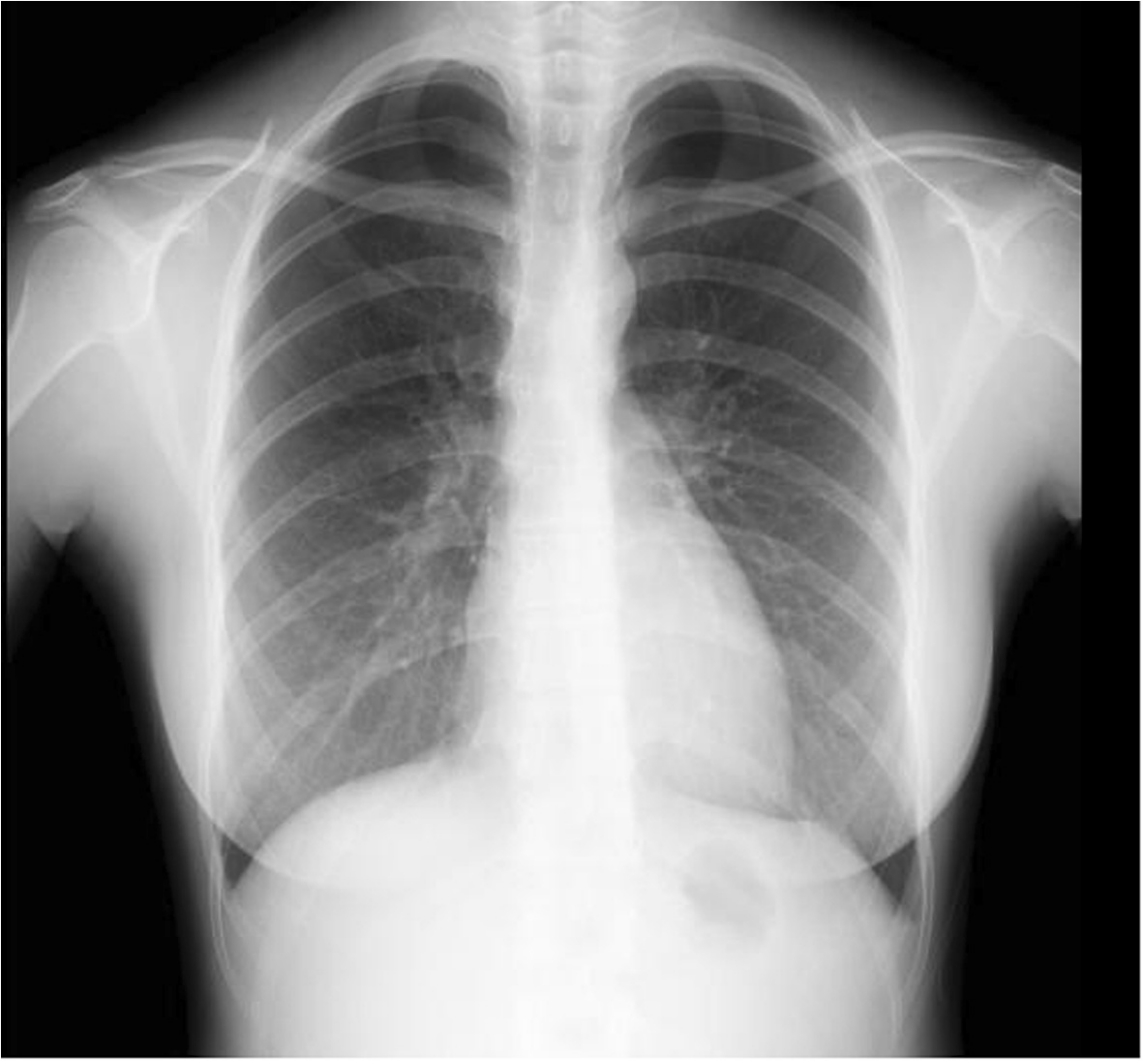
**Chest X-ray showed bilateral pneumothorax in a health screening.**

A 25-year-old woman visited our hospital because of bilateral pneumothorax detected on a chest X-ray taken during a health screening (Figure 1). She had neither chest-related symptoms nor a rash on her body or face. She was a non-smoker and had no history of major diseases. However, she had a family history of spontaneous pneumothorax that included her father, paternal uncle, and paternal aunt.

**Figure 2:**
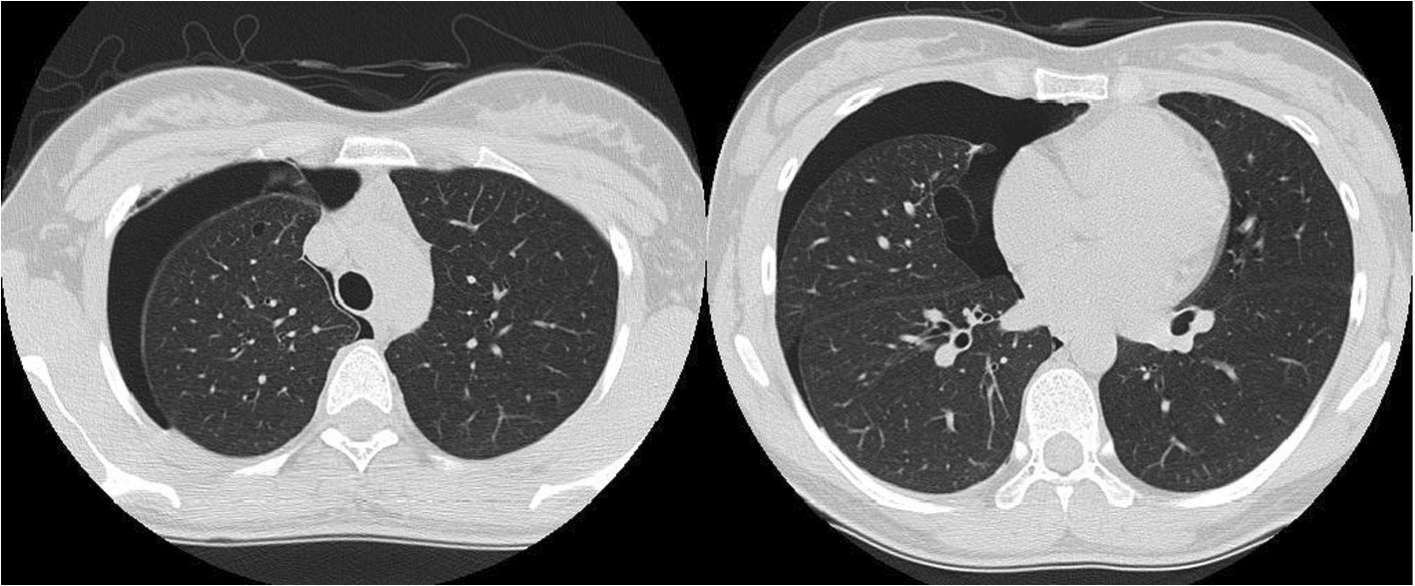
**Chest CT showed right-sided pneumothorax and multiple cystic lesions in both of the patient's lungs.** Adhesion and thickened visceral pleura were found.

A chest computed tomography (CT) scan taken at our hospital showed right-sided pneumothorax as well as almost healed left-sided pneumothorax (Figure 2). It also showed multiple cystic lesions throughout both lungs.

We performed video-assisted thoracic surgery (VATS) on the patient's right lung. Repetition of pneumothorax was suspected because pleural adhesion and thick pleura were found in her thorax. Many bullas were found at the surface of the lung; therefore, resection and ablation of bullas were performed, followed by stapling of the staple lines and reinforcement of ablated sites using a polyglycolic acid (PGA) sheet and fibrin glue. The patient's post-operative course was uneventful.

One month after the operation, she visited our hospital again complaining of respiratory discomfort. Chest X-ray and CT scan showed left-sided pneumothorax. Therefore, we performed VATS on the left lung. Again, many minuscule bullas appeared throughout the whole lung, and pleural adhesion and thick pleura were observed as before in the right lung. The surgery was performed following the same methods used for the right lung. Again, the patient's post-operative course was uneventful, and the recurrence of pneumothorax was not observed in either lung at 6 months after the second operation.

**Figure 3:**
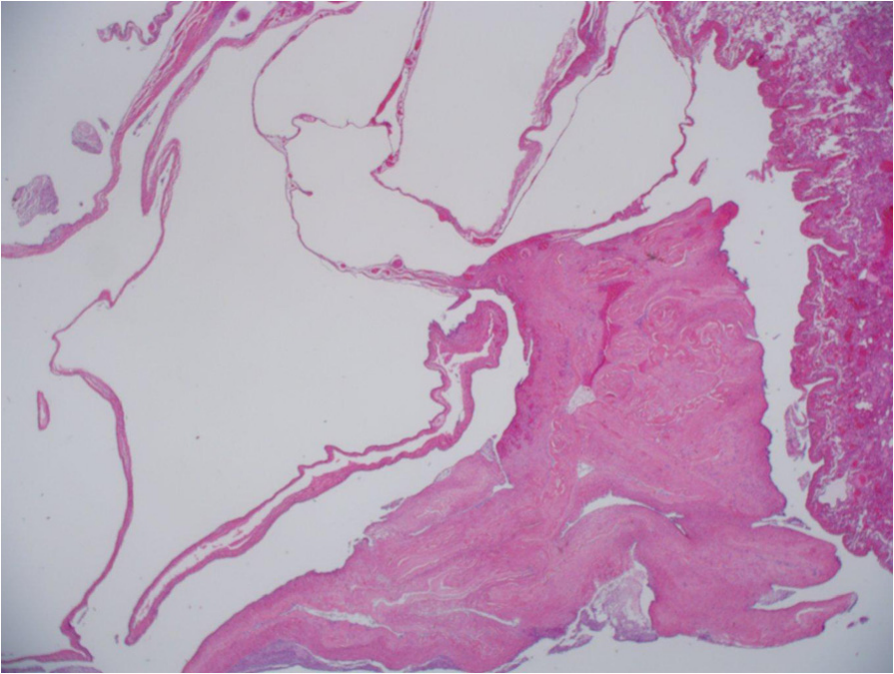
**Histopathological examination.** The inner surface of cysts was lined by epithelial cells, and the cysts occasionally contained internal septa consisting of alveolar walls.

Histopathological examination showed that the inner surfaces of cysts were lined with epithelial cells, and the cysts occasionally contained internal septa consisting of alveolar walls (Figure 3). There were no special pathological findings of lymphangioleiomyomatosis (LAM) or catamenial pneumothorax, including LAM cell clusters or formation of chocolate-like endometriotic cysts.

**Figure 4:**
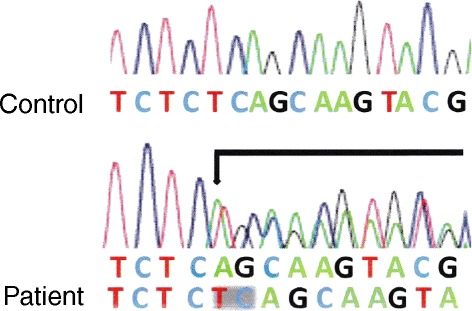
**Sequence diagram.** Sequence diagram of exon 12 of the BHD gene shows the two deletions of bases compared with the control.

Because the patient was young and a non-smoker with prominent family history of pneumothorax and multiple cystic lesions were observed throughout her whole lung, we suspected congenital disease and particularly BHD, despite the absence of fibrofolliculomas. We presented this case to a special research team focusing on BHD, which was formed as a specialized disease treatment research program by the Japanese government. The patient was diagnosed as having BHD via a DNA analysis, which showed two deletions of bases in exon 12 of the BHD gene (Figure 4). No renal tumors were observed by abdominal ultrasonography.

## Discussion

Multiple pulmonary cysts with repeated pneumothorax, renal tumors, and fibrofolliculomas are the three main symptoms of BHD. In Europe and the United States, BHD is well known, and treatment approaches are established. However, in East Asia, particularly in Japan, BHD is not yet well known, and this may be related to differences in major symptoms of BHD among different ethnic backgrounds.

Fibrofolliculoma of the skin with three characteristic symptoms can be detected only in 20% to 27% of the BHD cases in Japan [4-6], whereas in Europe and the United States, these symptoms are present in approximately 90% of the BHD patients [7-9]. In contrast, multiple pulmonary cysts with repeated pneumothorax are seen in approximately 90% of the BHD cases in Japan [5], whereas pneumothorax occurs in only 34% of the BHD cases in Europe and the United States [7]. Furuya et al. say that some cysts have veins into the cystic spaces, and most of the epithelium lining the cyst appears to be friable and some epithelial tissue exfoliates from the cyst wall, and more, some remaining tissue appears thin. These complex structures may be often associated with chronic inflammation and develop rupture and pneumothorax [4]. In the present case, we ruled out LAM or catamenial pneumothorax via histological examination, and then we considered the possibility of BHD because the patient was young and a non-smoker with a family history of pneumothorax who presented with multiple cysts throughout the whole lung and experienced repeated pneumothorax.

In the present case, BHD mutations were identified at exon 12 (c.1379_1380 delTC) of the FCLN gene, which is a relatively rare pattern [2,7]. Toro et al. reported that there is no relationship between the site of such a germline mutation and clinical symptoms of BHD [2]. However, the number of BHD cases analyzed in their study was small, and we need an analysis of more cases to clarify the relationship between the gene mutation pattern and clinical symptoms.

In this case, we performed bullasectomy for large bullas and ablation to eliminate small bullas. Small bullas were contracted sufficiently by heat ablation using large ball chip for electrocoagulation, and its power was 15 W in spray coagulation mode. In addition, we reinforced the visceral pleura using PGA seats and fibrin glue. The effectiveness of visceral pleura covering by absorbable sheets, including PGA and oxidized regenerated cellulose, against repeated pneumothorax has been reported more often in recent years. Although it is unclear whether these surgical methods are effective for avoiding recurrence of pneumothorax in BHD patients, studies including greater number of cases are needed to establish the optimal treatment for BHD-related pneumothorax.

Because renal tumors are strongly associated with the prognosis of BHD patients, early detection and early treatment of the renal tumors are quite important. Pavlovich et al. reported that renal tumors are present in 27% of the BHD patients, and the mean age at the occurrence of renal tumors is 50.4 years [9]. In Japan, renal cancer is detected in 21% of the BHD patients [4]. This percentage is similar to those in Europe and the United States. The patient in the present case had not developed any renal tumors at the last follow-up before the preparation of this report. However, the patient and her family members should be routinely screened for renal tumors going forward.

In the present case, the inner surface of pulmonary cysts was lined by epithelial cells, and the cysts occasionally contained internal septa consisting of alveolar walls. Furuya et al. reported that these histological findings are often seen in BHD [4]. In contrast, another investigator said that there are no specific histological findings related to BHD [10]. Therefore, it is commonly difficult to diagnose BHD only on the basis of histopathological findings of resected bullas [8]. However, it is important to suspect BHD at the time of treatment of pneumothorax patients, particularly when the patient has a family history of pneumothorax, has multiple pulmonary cysts, and experiences repeated pneumothorax, even if fibrofolliculomas are not seen in their skin.

## Conclusions

We report a case of a young Japanese woman in whom asymptomatic bilateral pneumothorax was detected incidentally during a health screening, which led to the diagnosis of BHD, and who did not present fibrofolliculomas. When we encounter patients who have experienced repeated pneumothorax, particularly those who have family history of pneumothorax and/or multiple pulmonary cysts throughout the entire lung, we should consider the possibility of BHD and perform the relevant genetic analysis.

## Consent

Written informed consent was obtained from the patient for publication of this case report and any accompanying images. A copy of the written consent is available for review by the Editor-in-Chief of this journal.

## Abbreviations

BHD: Birt-Hogg-Dubé syndrome
CT: computed tomography
FCLN: folliculin
LAM: lymphangioleiomyomatosis
PGA: polyglycolic acid
VATS: video-assisted thoracic surgery
